# Comorbidity, age, race and stage at diagnosis in colorectal cancer: a retrospective, parallel analysis of two health systems

**DOI:** 10.1186/1471-2407-8-345

**Published:** 2008-11-25

**Authors:** S Yousuf Zafar, Amy P Abernethy, David H Abbott, Steven C Grambow, Jennifer E Marcello, James E Herndon, Krista L Rowe, Jane T Kolimaga, Leah L Zullig, Meenal B Patwardhan, Dawn T Provenzale

**Affiliations:** 1 Department of Medicine, Duke University Medical Center, Durham, USA; 2 Department of Palliative and Supportive Services, Flinders University, Australia; 3 Center for Health Services Research in Primary Care, Durham Veterans Administration Medical Center, Durham, USA; 4 Department of Biostatistics and Bioinformatics, Duke University Medical Center, Durham, USA; 5 Cancer Center Biostatistics, Duke University Medical Center, Durham, USA; 6 Duke Center for Clinical Health Policy Research, Duke University Medical Center, Durham, USA

## Abstract

**Background:**

Stage at diagnosis plays a significant role in colorectal cancer (CRC) survival. Understanding which factors contribute to a more advanced stage at diagnosis is vital to improving overall survival. Comorbidity, race, and age are known to impact receipt of cancer therapy and survival, but the relationship of these factors to stage at diagnosis of CRC is less clear. The objective of this study is to investigate how comorbidity, race and age influence stage of CRC diagnosis.

**Methods:**

Two distinct healthcare populations in the United States (US) were retrospectively studied. Using the Cancer Care Outcomes Research and Surveillance Consortium database, we identified CRC patients treated at 15 Veterans Administration (VA) hospitals from 2003–2007. We assessed metastatic CRC patients treated from 2003–2006 at 10 non-VA, fee-for-service (FFS) practices. Stage at diagnosis was dichotomized (non-metastatic, metastatic). Race was dichotomized (white, non-white). Charlson comorbidity index and age at diagnosis were calculated. Associations between stage, comorbidity, race, and age were determined by logistic regression.

**Results:**

342 VA and 340 FFS patients were included. Populations differed by the proportion of patients with metastatic CRC at diagnosis (VA 27% and FFS 77%) reflecting differences in eligibility criteria for inclusion. VA patients were mean (standard deviation; SD) age 67 (11), Charlson index 2.0 (1.0), and were 63% white. FFS patients were mean age 61 (13), Charlson index 1.6 (1.0), and were 73% white. In the VA cohort, higher comorbidity was associated with earlier stage at diagnosis after adjusting for age and race (odds ratio (OR) 0.76, 95% confidence interval (CI) 0.58–1.00; p = 0.045); no such significant relationship was identified in the FFS cohort (OR 1.09, 95% CI 0.82–1.44; p = 0.57). In both cohorts, no association was found between stage at diagnosis and either age or race.

**Conclusion:**

Higher comorbidity may lead to earlier stage of CRC diagnosis. Multiple factors, perhaps including increased interactions with the healthcare system due to comorbidity, might contribute to this finding. Such increased interactions are seen among patients within a healthcare system like the VA system in the US versus sporadic interactions which may be seen with FFS healthcare.

## Background

CRC is the second most common cancer in Europe and the fourth most common cancer in the United States (US) [1,2]. CRC is the second leading cause of cancer death in both Europe and the US [1,2]. Advanced age is the most significant risk factor for diagnosis of CRC, as the vast majority of patients are diagnosed when 65 and older with a peak incidence of 415.9 cases per 100,000 in those 85 and older [3]. Despite advances in screening and treatment, stage at diagnosis remains the most important predictor of mortality. The five-year survival rate for localized disease is 90.4%, but only 39% of CRCs are diagnosed at this early stage [3]. Of those diagnosed with metastatic disease, less than 10% are still alive after 5 years [3].

**Figure 1:**
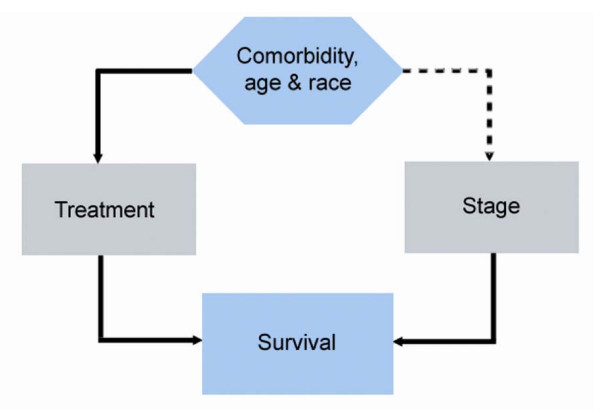
**Relationship between stage at diagnosis and comorbidity, age, and race**. Comorbidity, age, and race are known to influence the delivery of stage-appropriate chemotherapy, and thereby influence survival. We hypothesized (dotted line) that comorbidity and age also influence survival by determining stage at diagnosis.

Since stage at diagnosis plays a significant role in CRC survival, understanding which factors contribute to a more advanced stage at diagnosis is vital to improving overall survival. Potential factors include comorbidity, age, and race. These factors are known to influence cancer outcomes at least in terms of receipt of standard therapy, which in turn, likely influences survival [4-10]. While the relationship between delivery of treatment and these factors has been established, the association between comorbidity, age, race, and stage at diagnosis of CRC is less clear (Figure 1).

Access to health care and health insurance has been associated with disparities in cancer survival and late stage of diagnosis [11,12]. The purpose of this analysis is to describe the association between stage at diagnosis and comorbidity, age, and race in patients with CRC, and the influence that the healthcare system has on this association. Two US health systems, fee for service (FFS) and the Veterans Administration (VA), were selected because they allow assessment of predictors of stage of diagnosis that may be unique to the health system versus the population. Healthcare in the United States is provided through multiple parallel and overlapping systems roughly divided between commercial, public, and uninsured/self-pay programs [13]. FFS represents the context where patients usually have some form of commercial health insurance to cover most healthcare costs, access to specialist care is more easily obtained, and primary care may or may not be promoted depending upon the type of insurance provider (e.g. managed care, preferred provider organization) [13]. The VA system provides care to military service veterans and their families; it is a closed system with access to primary care providers who act as gatekeepers to specialist care. The VA system is generally regarded as a US healthcare system where access to care is less influenced by ability to pay [14]. We hypothesized that patients with greater comorbidity, older age, and white race would be more likely to be diagnosed with early-stage CRC, due to more frequent contact with the healthcare system. We hypothesized that these differences would be most prominent in the VA system where access to high-quality primary care is encouraged [15,16].

## Methods

### Study Population

The first patient cohort was derived from patients with CRC enrolled in the Cancer Care Outcomes Research and Surveillance Consortium (CanCORS), a cohort study to evaluate the care of patients with newly diagnosed lung or CRCs recruited in geographically diverse populations and health care systems [17]. Eligible patients were ≥ 21 years old and were diagnosed with colorectal adenocarcinoma within 3 months of enrollment. Approximately 4,920 patients with CRC have been enrolled in CanCORS. Using the CanCORS database, we identified patients with CRC diagnosed and treated at 15 Veterans Administration (VA) hospitals from 2003 (cohort initiation) to 2007. Of the approximately 4,920 CRC patients enrolled in the CanCORS study, 477 received treatment in the VA health system. Of those, 342 had complete chart abstractions at the time of this analysis, met inclusion criteria (as described above), and were included in this analysis. Informed consent was obtained from all living patients and a HIPAA decedent consent waiver was obtained for deceased patients.

The second patient cohort was taken from community and academic FFS practices in the Southeastern US, as part of a larger study designed to examine regional treatment patterns for metastatic CRC [18]. Eligible patients were adults diagnosed with metastatic CRC (recurrent or primary) from January 2003 to June 2006, and treated at one of ten participating community or academic sites. Medical records for patients treated prior to 2003 for non-metastatic CRC were also included, which allowed for assessment of stage at diagnosis. Of 743 charts initially screened from local Tumor Registry lists, 340 met eligibility criteria and had abstracted charts available at the time of this analysis. Informed consent was waived by the Institutional Review Board for this patient cohort. The research presented was conducted in accordance with the principles of the Helsinki Declaration [19].

### Data Collection

We conducted retrospective review of patient records selected from two distinct patient populations, as noted above. Data were abstracted pertaining to race, cancer diagnosis, stage, age at diagnosis, and presence of comorbid illnesses. Stage at diagnosis was determined from review of pathology reports in concert with radiology and physician notes. The TNM staging system was used. Stage at diagnosis was dichotomized to non-metastatic (defined as ≤ stage III, localized, or regional) and metastatic due to sample size limitations. Age at diagnosis was recorded in years for each patient. Race was dichotomized to white and non-white.

The presence of comorbid conditions was assessed based on chronic illnesses identified in the Deyo adaptation of the Charlson comorbidity index [20]. The Charlson index was developed as a reproducible measure of the prognostic impact of comorbid illnesses [21]. The index has been validated in oncology populations and has since been adapted for use with administrative databases [20]. Patient medical records were abstracted for chronic medical conditions already established at the time of CRC diagnosis (Table 1). The conditions were weighted and patients were assigned a score in accordance with the Charlson index [21]. The comorbidity score was modelled as an integer-valued quantitative variable.

**Table 1:** Charlson Comorbidity Index conditions and weights

**Weight**	**Condition**
**1**	Myocardial infarction, Congestive heart failure, Peripheral vascular disease, Cerebrovascular disease, Dementia, Chronic pulmonary disease, Rheumatologic disease, Peptic ulcer disease, Chronic liver disease (mild), Diabetes (uncomplicated)

**2**	Hemiplegia, Renal disease (moderate or severe), Diabetes with end organ damage, Solid tumor without metastasis, Leukemia or lymphoma

**3**	Liver disease (moderate or severe)

**6**	Metastatic solid tumor, AIDS

Weights are determined by 1-year relative risk of mortality associated with presence of the comorbidity. Comorbidity index per patient is calculated by the addition of weighted scores as determined by the Charlson index [21].

### Data Analysis

The primary goal of the analysis was to examine associations between stage at diagnosis and an *a priori *set of variables (comorbidity, age, and race) using logistic regression modelling in cohorts selected from two different patient populations. The two cohorts were not jointly analyzed due to differences in patient characteristics from both studies and overall study design. Results are presented for each cohort as unadjusted and adjusted odds ratios with associated 95% confidence intervals and p-values. Odds ratios and associated confidence intervals are also presented graphically to facilitate understanding of the similarities and differences in the patterns of association between the cohorts [22]. Linearity assumptions of continuous covariates (age and comorbidity) were assessed using graphical techniques and no evidence of systematic non-linearity was found. Statistical analyses were performed using SAS for Windows Version 9.1 (SAS Institute, Cary, NC). Two-sided p-values at the standard 0.05 level were used to determine statistical significance.

## Results

### Patient Characteristics

Six hundred eighty-two patients met the eligibility criteria for this analysis (Table 2). The VA cohort was older than the FFS cohort (mean age ± standard deviation, 67 ± 11 years in the VA versus 61 ± 14 years in FFS). As expected, the VA cohort was predominantly male (98%) as opposed to the FFS cohort, which was evenly divided by gender. More VA patients than FFS patients had a Charlson score ≥ 3 (11% VA versus 7% FFS). Both cohorts were predominantly white. The FFS cohort was comprised of a higher percentage of patients with stage IV disease at diagnosis due to original intent and design of the study from which this patient cohort was drawn [18].

**Table 2:** Characteristics of patients in both VA and fee-for-service settings.

**Characteristic**	**VA patients^1 ^(n = 342)**	**Fee-for-service patients^2 ^(n = 340)**
Stage at diagnosis (%)		
Metastatic	27	77
Non-metastatic	73	24
Charlson comorbidity score (Mean ± SD)	2.0 ±	± 1.0
Age in years (Mean ± SD)	67 ±	± 14
Gender (%)		
Male	98	48
Female	1.7	52
Race (%)		
White	63	73
Non-white	37	27

^1^Missing value summary for VA: race (n = 6); ^2^Missing value summary for FFS: Charlson (n = 14), age (n = 47), gender (n = 11), race (n = 45),

### Logistic Regression

The statistical results from the logistic regressions are summarized in Table 3 and discussed below.

**Table 3:** Association of metastatic stage of colorectal cancer diagnosis with comorbidity, age, and race in two patient cohorts

**Characteristic**	**VA**		**FFS**	
	**OR (95% CI)**	**p-value**	**OR (95% CI)**	**p-value**
Comorbidity				
Unadjusted	0.78 (0.61, 1.00)	0.04	1.08 (0.82, 1.42)	0.59
Adjusted	0.76 (0.58, 1.00)	0.045	1.09 (0.82, 1.44)	0.57
Age (≥ 70 years)				
Unadjusted	0.98 (0.79, 1.23)	0.88	0.97 (0.80, 1.18)	0.76
Adjusted	1.08 (0.85, 1.36)	0.54	0.96 (0.78, 1.17)	0.67
Race (white)				
Unadjusted	0.80 (0.49, 1.31)	0.39	1.12 (0.61, 2.08)	0.72
Adjusted	0.83 (0.51, 1.37)	0.47	1.12 (0.60, 2.09)	0.72

Adjusted odds ratios were calculated from a multivariable logistic regression model that included Charlson comorbidity score (modelled as an integer-valued quantitative variable), age at diagnosis (expressed in years), and race (white vs non-white). The unadjusted and adjusted odds ratios, shown here with associated 95% confidence intervals and p-values, are for a single unit increase in comorbidity, a decade increase in age, and the comparison of white vs. non-white for race. Final n for VA adjusted model = 336 (91 metastatic, 245 non-metastatic), final n for FFS adjusted model = 270 (198 metastatic, 72 non-metastatic).

### Influence of Comorbidity

In the VA cohort, higher comorbidity was associated with non-metastatic stage at diagnosis. This relationship was maintained after adjusting for age and race (adjusted odds ratio (OR) 0.76, 95% confidence interval (CI) 0.58–1.00). In the FFS cohort, however, no relationship was seen between comorbidity and stage at diagnosis (adjusted OR 1.09, 95% CI 0.82–1.44).

### Influence of Age

In both the VA and FFS cohorts, no evidence was found of an association between stage at diagnosis and age. The CI's for the OR's in both populations span 1.0 and overlap each other, indicating that older age was not associated with stage at diagnosis. These assertions hold whether or not the results were adjusted for comorbidity and race.

### Influence of Race

The influence of race was not statistically significant for either cohort. The unadjusted and adjusted confidence intervals for the odds ratios broadly spanned 1.0 for both cohorts.

## Discussion

This analysis provides a health system-based perspective on the association between stage of CRC diagnosis and comorbidity, race, and age. Higher comorbidity was associated with non-metastatic stage at diagnosis in the VA cohort, but not in the FFS cohort. Neither age nor race was associated with stage at diagnosis. The findings may be to due to multiple factors, some of which were unmeasured in this analysis.

A critical, comorbidity-related concern in cancer outcomes relates to how comorbidity influences survival among cancer patients. Comorbidity is already known to negatively influence the delivery of stage-appropriate treatment (Figure 1), and not receiving appropriate therapy will negatively influence survival outcomes [23-29]. As the population ages, the increasing burden of comorbid illness will likely play a greater role in cancer treatment-related toxicity and outcomes. Higher comorbidity has been shown to broaden the survival disparity between blacks and whites with CRC [24]. Both comorbidity and advanced age have been associated with worse outcomes after surgery for CRC [23,25,26,29], decreased referral to a medical oncologist [23,28], and incomplete courses of adjuvant chemotherapy [27,30]. By best understanding where in the cancer care continuum comorbidity, age, and race exert their greatest influence, we can better determine how to improve care for patients with comorbid illnesses and CRC.

In the VA cohort, where increasing comorbidity was associated with earlier stage at diagnosis, patients with multiple comorbid illnesses might have more frequent health care contact and thus benefit from the greater clinical scrutiny it provides. Evidence demonstrates that VA patients are more likely to experience higher quality primary care than FFS patients; a corollary to increased access to primary care is an increase in routine chronic and preventive medical care that may be overlooked in commercial health systems with less access to primary care. In non-VA populations, FFS health care might lead to erratic interaction with the health care system as determined by ability to pay. For example, VA patients have been shown to do better with diabetes and hyperlipidemia control than patients in commercial health systems [15]. VA patients are more likely to report receiving diabetes education than those covered by private insurance [31]. Hence, the increased medical scrutiny inherent when a person has multiple comorbidities coupled with a health system that provides easy access to primary care providers might ensure delivery of age-appropriate screening or might lead to the incidental diagnosis of cancer through blood tests or studies ordered for other purposes.

Other factors may explain the relationship between comorbidity and stage observed in the VA cohort and not in the FFS cohort. Most notably, the difference might be due to differences in the two cohorts. The VA cohort was older, predominantly male, and carried a higher burden of comorbid illnesses than the FFS cohort. This difference between the VA and non-VA cohorts in our analysis is representative of the VA and non-VA populations as a whole [32-35]. The association might be influenced by the comorbid conditions themselves, leading to a varied influence of comorbidity on stage based more upon population and less on health system. For example, symptoms attributed to comorbid illnesses might actually be arising from a neglected, evolving malignancy [36]. Second, the pathophysiology of the comorbid illness or its treatment might contribute to the development or progression of the cancer. For example, a growing body of data has demonstrated an association between chronic insulin therapy and development of CRC among patients with type II diabetes mellitus [37]. Such factors might have diminished (in the VA group) or nullified (in the FFS group) the effect of comorbidity on stage at diagnosis.

Another factor may have been the definition of the FFS cohort. Eligible patients had metastatic disease during 2003–2006, although their prior non-metastatic period was also considered. As a result, the cohort was enriched with patients who were stage IV at diagnosis. This group may have less screening and healthcare involvement at baseline, and the impact of comorbidity status may have been diluted. Nonetheless, this group still had a quarter of patients who were non-metastatic at diagnosis.

In our analysis, race and age were not influential factors in the stage of CRC diagnosis. Age was not influential likely because numeric age is less important clinically when the degree of comorbidity is measured. In other words, older patients without significant comorbidity should have similar clinical outcomes than relatively younger patients without comorbidity. For instance, when older patients receive chemotherapy, they are able to tolerate and respond to treatment as well as their younger counterparts [38-41]. The lack of interaction with race and stage at diagnosis might be due to a similar process. Bach et al found that after controlling for population mortality (non-cancer related death), the difference in cancer-related mortality between blacks and white was diminished [24]. The racial differences in stage at diagnosis might be reduced when adjusted for comorbidity. We did not, however, account for differences in socioeconomic status, which might have also influenced the role of race.

In framing the significance of our findings, the limitations of this study must be discussed. First, in terms of categorizing comorbidity, we chose to use the Charlson index, which has been validated in the oncology setting. Despite being updated, the index was created in 1984 and based on admissions to a hospital over a one-month period [20,21], which brings into question the generalizability of the index to cancer diagnosis and treatment today [42]. The Charlson index does not grade severity of comorbidity, nor does it capture functional disability. It might not be measuring comorbid conditions that are most relevant to a cancer population. On the other hand, the ease of use, reliability, and content validity of the index make it a reasonable choice [42].

Second, asymptomatic screening was not addressed as a variable in this study as patient screening information was not available for both patient cohorts. Patients with a high comorbidity index might be less likely to undergo cancer screening due to an increased risk for non-cancer-related mortality. If this were the case, the relationship between comorbidity and stage at diagnosis might be less important than the relationship between comorbidity and receipt of screening, as delayed screening could contribute to later stage at diagnosis. However, studies conducted in both VA and non-VA populations have shown that patients are screened for CRC regardless of comorbidity status, thereby suggesting that no relationship exists between degree of comorbidity and rates of asymptomatic screening [43-46]. If screening rates are not influenced by comorbidity, then stage at diagnosis becomes the next most important variable for investigation. Furthermore, if younger, healthier patients are more likely to be screened for CRC, then this analysis should have found that younger, healthier patients present with non-metastatic disease, and older, sicker patients present with metastatic disease. It did not.

This analysis does not explicitly address access to screening studies such as colonoscopy. Multiple factors have been associated with access or adherence to colorectal cancer screening, including age, education, insurance status, and a usual source of care [47]. Our presented data cannot address these characteristics completely, though the literature supports the assumption that VA patients enjoy greater access to many aspects of primary care than Medicare fee-for-service and privately insured patients [15,16,31]. While specific data is not available for our sample, the rate of CRC screening with endoscopy is fairly equivalent between VA and non-VA populations [48,49].

This study has strengths that overcome limitations of prior studies. First, we provide consistent data from two distinct health care systems. Our study is the first to examine the relationship between comorbidity, age and stage at diagnosis in the VA health system. As receipt of care in this system is less dependent on ability to pay, it serves as a useful control for the inability to access appropriate cancer care [50]. Second, this study does not dichotomize comorbidity, but models it as an integer-valued quantitative variable (0–≥ 3) with Charlson score ≥ 3 equated with severe comorbidity. A score of ≥ 3 predicts a significantly greater risk of non-cancer-related mortality over a one-year period [21]. A prior study demonstrated a slightly higher prevalence of comorbidity in patients diagnosed with early-stage (Dukes' A) CRC, but this study dichotomized comorbidity with a cut point of ≥ 1 [51]. A study by Gonzalez et al found the opposite: patients with any comorbid condition (Charlson score ≥ 1 vs. 0) were more likely to be diagnosed at late-stage, though interestingly, this finding did not persist with a Charlson score of ≥ 2 [52]. Based on these findings, we chose not to dichotomize comorbidity. This more quantitative use of the comorbidity index, as opposed to simply measuring presence versus absence of any comorbidity, might provide a more clinically-useful model when assessing the role of a comorbidity index on the diagnosis, treatment, and outcomes of cancer. A third strength of our study lies in our ability to measure comorbidity. Studies examining stage with larger cohorts obtained from cancer registries such as the Surveillance, Epidemiology, and End Results (SEER) database do not have access to comorbidity data.

Our exploratory results help to focus future efforts on improving CRC outcomes. As the association between comorbidity and cancer outcomes becomes clearer, future prospective studies should examine where in the patient's cancer care trajectory a specific comorbid illness might impact outcomes [53]. For example, among patients with breast cancer, studies have shown that only certain comorbid illnesses (such as diabetes) contribute to a later stage at diagnosis, while others (such as cardiovascular disease) do not [54]. Our exploratory findings should encourage further investigation into how comorbid illnesses, and not just comorbidity indices, impact diagnosis, treatment, and survival. If patients are even more likely to be diagnosed at early stage in the setting of comorbid illness, it becomes even more important to develop CRC treatment strategies that accommodate comorbidities. Future analysis will ask similar questions of the complete, multi-health system CanCORS sample, which includes approximately 10,000 lung and colorectal cancer patients. Socioeconomic characteristics, including income and education, should also be explored in conjunction with comorbidity. These factors cannot be appropriately investigated through SEER-Medicare linked datasets but can be explored via CanCORS.

As cancer treatment improves, more patients are living longer. As a result, more attention needs to be paid to the role of comorbidity and the patient's overall health as comorbidity might actually play a greater role in survival than the cancer, itself. Studies have shown that some cancer patients might be less likely to adopt diet, exercise and other healthy lifestyle changes after a cancer diagnosis [55]. Future interventional studies among cancer patients might investigate how better controlling health habits and comorbid illnesses might improve health-related quality of life and cancer-related survival outcomes. Data from this study suggests that the role of the health system and primary care provider may be an important influence in timing of diagnosis of CRC; similarly, access to primary and routine healthcare will be important for maintenance of health in the cancer survivorship period.

Currently, oncologists are treating older or frailer patients based on clinical trial data from younger, healthier patients [56-58]. If a better understanding is gained as to the role of comorbidities in cancer outcomes, patients with greater comorbidity and advanced age could be enrolled and evaluated separately in clinical trials in order to understand how they truly benefit from advances in cancer therapy. Such diversification of the clinical trial population would produce clinical trial data that is more representative of the typical cancer patient who might not be otherwise eligible for a clinical trial but might still benefit from some form of treatment.

## Conclusion

In this analysis of patients with CRC treated in two distinct health systems, we found that higher comorbidity is related to earlier stage of CRC diagnosis for VA patients. This finding might be the result of many factors, some unmeasured in this analysis. One factor might be the way care is delivered within certain US health systems, where access to care is less related to ability to pay and primary care is promoted. Future studies should be designed to focus on the impact of specific comorbidities on both stage at diagnosis and survival outcomes.

## List of abbreviations

CanCORS: Cancer Care Outcomes Research and Surveillance Consortium; VA: Veterans Administration; FFS: fee-for-service; OR: odds ratio; CI: confidence interval; US: United States; SEER: Surveillance, Epidemiology, and End Results.

## Competing interests

The fee-for-service portion of this chart review study was funded through an Outcomes Research service agreement with Pfizer, Inc., as an assessment of patterns of care among patients with metastatic CRC. Pfizer, Inc. does not have access to individual data nor authority to prohibit publication of results. None of the authors have a conflict of interest to report relevant to this Pfizer funding relationship.

## Authors' contributions

SYZ conceived of this analysis, participated in its design, and drafted the manuscript. APA contributed data from the FFS patient cohort, participated in study conception and design, assisted in data interpretation, and helped draft the manuscript. DHA performed the data analysis and helped draft the manuscript. SCG designed and directed the statistical analysis, and helped draft the manuscript. JEM assisted with data management. JEH contributed to developing the analytic plan. JTK assisted with data management and participated in study design. LLZ assisted with data management and analytic design. MBP participated in analytic design, data management, and drafting of the manuscript. DTP contributed to analysis conception, design, data interpretation, and helped draft the manuscript.

**Figure 2:**
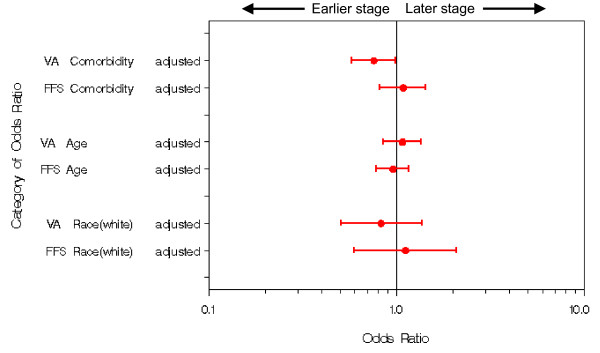
**Comorbidity, Age, and Race: Impact on Stage**. The impact of comorbidity, age, and race on stage at diagnosis is measured by the adjusted odds ratios of these factors. The odds ratios, shown here with 95% confidence intervals, are for a single unit increase in comorbidity, a decade increase in age, and the comparison of white vs. non-white for race. The relationship between comorbidity and stage among VA patients is statistically significant, but the upper bounds of the CI rounds to 1.0 (0.998).

## Pre-publication history

The pre-publication history for this paper can be accessed here:


